# Opposite poles: A comparison between two Spanish regions in health-related quality of life, with implications for health policy

**DOI:** 10.1186/1471-2458-10-576

**Published:** 2010-09-26

**Authors:** Juan Oliva-Moreno, Néboa Zozaya, Beatriz G López-Valcárcel

**Affiliations:** 1 Departamento de Análisis Económico and Seminario de Investigación en Economía de la Salud, University of Castilla La Mancha, and CIBER Epidemiología y Salud Pública (CIBERESP), Toledo, 45071, Spain; 2 Seminario de Investigación en Economía de la Salud. University of Castilla La Mancha, Toledo, 45071, Spain; 3 Quantitative Methods in Economics, University of Las Palmas de Gran Canarias. 35017 Las Palmas de Gran Canarias. Spain

## Abstract

**Background:**

Although health is one of the main determinants of the welfare of societies, few studies have evaluated health related quality of life in representative samples of the population of a region or a country. Our aim is to describe the health-related quality of life of the inhabitants of two quite different Spanish regions (Canary Islands and Catalonia) and to compare the prevalence of health problems between age-sex groups.

**Methods:**

We use data obtained from the 2006 Health Survey of Catalonia and the 2004 Canary Islands Health Survey. With an ordinal composite variable measuring HRQOL we identify the association of characteristics of individuals with self-reported quality of life and test for differences between the regions.

**Results:**

The prevalence of problems in the five EQ-5 D dimensions increases with age and is generally higher for women than for men. The dimension with the highest prevalence of problems is "anxiety/depression", and there is noteworthy the extent of discomfort and pain among Canary Island women. Education, especially among the elderly, has an important effect on health-related quality of life.

**Conclusions:**

There are substantial structural and compositional differences between the two regions. Regional context is a significant factor, independent of the compositional differences, and the effects of context are manifest above all in women. The findings show the importance of disease prevention and the need for improving the educational level of the population in order to reduce health inequalities.

## Background

Health is one of the main determinants of the welfare of societies. Developed countries allocate a great amount of monetary and no-monetary resources to the care of their population's health, and the measurement of health and the analysis of the health determinants of a given population are important for health decision-makers and for the society at a large.

Traditionally, healthiness has been evaluated objectively based on observation or medical interventions that take into account general indicators such as life expectancy, mortality and prevalence of disease. However, these indicators have lost part of their predictive value in wealthy societies where disease tends to be chronic, the mortality rate is extremely low and life expectancy has reached new heights. This scenario calls for concepts and measures of health that are more dynamic, and a strictly biomedical model is being replaced by one including patients' assessments of their own health, or Self Rated Health (SRH)[1,2].

Subjective assessments and biological indicators are being combined to measure "health-related quality of life" (HRQOL), that is, the degree to which an individual's physical, social, functional and emotional well-being are impacted by health. During the last decades, quality of life has increased in importance as a health indicator for several reasons. It has become clear that mortality reduction cannot be the only objective in the face of chronic and degenerative diseases. We would not look forward to a world in which everyone lived to be 100, but most people had, say, neurodegenerative disease for the last thirty years of their lives. It has also become clear that the patient, rather than the physician, is often better able to judge his or her own state of health. And finally, new economic methods for evaluating health care technologies have facilitated and stimulated interest in subjective health and quality of life. As Sullivan[3] notes, "Medicine's epidemiological transition from acute to chronic disease is thus prompting an epistemological transition from primarily objective to primarily subjective evidence of health and health care effectiveness. Now some of the most important patient outcomes, like patient choices before them, are valid because they are subjective".

Differences between nations' self-rated health have been well documented. They can be attributed to the differing demographic composition of national populations, differences in context (due to culture, perception, etc.)[4] and differing national health organization and coverage, as a study comparing Canada and the United States suggests[5]. For countries with decentralized government, it is important to have a regional breakdown of self-rated heath in order to design appropriate public health policies. Spain, for instance, is divided into 17 autonomous regions, each with its own public health system.

The ordinal question on self-rated health included in many health surveys has been productive because despite its simplicity-- based on the single undifferentiated notion of healthiness--it is associated with the use of healthcare services [6-8]. Also, subjective health-related quality of life is a significant predictor of future functioning and mortality within countries [9-12] and among individuals with similar clinical conditions[7,13]. Some health surveys also ask interviewees how well they feel and whether their health has improved or worsened in the last months, and request a self-evaluation on different aspects of health status. One of the most popular descriptive systems for health-related quality of life is the EuroQol Group's EQ-5 D. This set of questions, which classes health states in five dimensions, has been used in specific groups and in the general population in several European countries, Japan, and in the US [14-19] and is commonly used in randomized clinical essays and in the evaluation of health care technologies. However, few studies have used the EQ-5 D questions to evaluate quality of life in representative samples of the population of a region or a country.

The aim of this study is to describe and compare the health-related quality of life of the inhabitants of two Spanish autonomous regions (the Canary Islands and Catalonia) in the first years of the twenty-first century using EQ-5 D data and to identify how characteristics of individuals are associated with self-reported quality of life. We compare the prevalence of health problems associated with each dimension of the EQ-5 D between age-sex groups of Catalans and Canarians. Then we model an ordinal composite variable measuring HRQOL separately for each age-sex group of Catalonia and the Canary Islands and test for differences between those regions.

The regions are quite different. Catalonia is on the Mediterranean in the northeast of Spain with 7.3 millions of inhabitants in January 2009 (15.9% of Spain's population), while the Canary Islands are in the Atlantic Ocean far to the southwest of Spain with 2.1 millions of inhabitants (4.5% of the total population of Spain). Per capita income is about 33% higher in Catalonia and standardized mortality per 100,000 inhabitants is lower (Table 1), particularly for ischemic disease and diabetes mellitus. Self-reported medically-diagnosed chronic conditions and risk factors like high blood pressure or cholesterol are substantially higher in Catalonia, but this may be because in Catalonia there are better diagnoses due to more effective primary care. We examine this aspect more closely by seeing if the impact on HRQOL of chronic conditions and other variables like level of education is the same in both regions. Our results could help regional administrations design geographically differentiated, citizen-oriented, health policies.

**Table 1:** Comparing age-adjusted mortality per 100,000 inhabitants in Catalonia and the Canary Islands 2006

Cause	Catalonia	Canary Islands
Cancer	157.8 (110.3)	161.6 (113.3)

Ischemic heart disease	43.7 (20.2)	73.3 (37.6)

Stroke	36.4 (11.3)	34.4 (12.7)

Diabetes Mellitus	11.6 (4.0)	30.7 (10.8)

All causes	512.3	575.2

In parentheses, adjusted mortality per 100,000 inhabitants at age younger than 75

Source: Spanish Ministry of Health, "Mortalidad por cáncer, por enfermedad isquémica del corazón, por

enfermedades cerebrovasculares y por diabetes mellitus en España" available in the webpage http://www.msc.es/

## Methods

Data used in the analysis of health-related quality of life were obtained from the 2006 Health Survey of Catalonia (CHS) and the 2004 Canary Islands Health Survey (CIHS). Both surveyed the general health of non-institutionalized adults (15 or more years of age in Catalonia, 16 years or more in Canary Islands) representative of each territorial unit. Participants in the surveys, therefore, spent most of the year residing in family dwellings that were their habitual residences. Individuals were excluded if they resided in collective homes or were hospitalized at the time of the survey. The sample sizes were 15,926 for Catalonia, and 4,282 for the Canary Islands. Both health surveys are official statistics and have been designed to get representative samples of the respective regional populations by sex, age groups and municipality stratum. The CHS is representative for each one of the 37 health areas existing in Catalonia with a maximum estimation error of ± 5%. The CHS was made jointly by the Department of Health and the Catalan Institute of Statistics. A random multistage stratified sample was obtained with two stages, first health region and second municipality. The CIHS design was a three- stage cluster sampling, and it is representative for each one of the seven islands. It was made jointly by the Canary Islands Health Service and the Institute of Statistics of Canary Islands. Both surveys were conducted by specialized interviewers through personal interviews. The questions included in both surveys were similar and comparable. Questionnaires and data of both surveys are openly available under request to Catalonia Department of Health and Canary Islands Health Service

Both surveys used the EQ-5 D schedule, which consists of five dimensions: mobility, personal care, usual activities, pain/discomfort and depression/anxiety. Health-related quality of life is measured by three possible answers in regard to functional state (no health problems, moderate health problems, extreme health problems) for each of the five dimensions. As a result there are 245 possible aggregate combinations (243 health states plus "unconsciousness" and "immediate death"). EQ-5 D has been developed as a valid instrument to measure self assessed health and it is widely used through the world [14,17].

Both surveys provide additional variables that could be associated with health-related quality of life: socio-demographic factors (age, gender, educational level), medically-diagnosed diseases or chronic conditions (vascular illness, rheumatic disease, digestive illness, mental illness, respiratory problems, diabetes mellitus, osteomuscular diseases), risk factors (body mass index, hypertension and abnormal cholesterol levels) and negative events surrounding health (undergoing hospitalization) and lifestyle (smoking, alcohol intake).

Table 2 shows that Catalonia and the Canary Islands differ in educational levels (higher in Catalonia) and in prevalence of vascular diseases), respiratory diseases and digestive diseases. On the contrary, osteomuscular problems are higher in Catalonia. Obesity is more prevalent in the Canary Islands than in Catalonia. Although diagnosed cardiovascular risk factors are more frequent in Catalonia than in the Canary Islands, it may be that real cardiovascular risk is higher in the Canary Islands, as rates of cardiovascular mortality suggest, but that risks are underdiagnosed.

**Table 2:** Demographic and clinical characteristics of study population

	Catalonia (CHS 2006)			Canary Islands (CIHS 2004)		
	**16-44**	**45-64**	**65 and** **older**	**16-44**	**45-64**	**65 and/b>** **older**

Good health: no problems in the five EQ-5 D dimensions	73.1%	50.9%	25.5%	66.4%	43.1%	31.8%

Fair/good health: 1 moderate problem in one of the five EQ-5 D dimensions	16.2%	19.6%	17.8%	18.3%	21.0%	19.1%

Fair/bad health: 2 or 3 moderate problems in the five EQ-5 D dimensions	6.3%	14.4%	22.9%	8.4%	18.1%	21.2%

Bad health: more than 3 moderate problems and/or any serious problem in the five EQ-5 D dimensions	4.6%	15.0%	33.8%	6.9%	17.8%	27.9%

Male/Female	51.9%/48.1%	49.2%/50.8%	41.6%/58.4%	51.0%.49.5%	50.5%/49.5%	44.0%/56.0%

No studies completed	2.4%	12.4%	46.1%	19.8%	46.7%	80.3%

Primary school completed	14.1%	28.8%	31.3%	31.1%	24.7%	12.1%

Secondary school completed	62.0%	43.0%	17.4%	25.5%	13.7%	3.2%

University completed	21.5%	15.7%	5.3%	23.6%	14.9%	4.3%

Vascular problems	2.5%	8.6%	30.2%	11.0%	27.3%	47.5%

Osteomuscular problems	30.1%	54.6%	74.3%	20.1%	43.5%	56.5%

Mental illness (depression/anxiety)	11.4%	23.8%	29.2%	12.0%	20.0%	21.9%

Respiratory problems	7.3%	9.2%	17.6%	4.0%	5.5%	9.6%

Digestive problems	4.4%	13.8%	0.0%	13.2%	24.0%	29.6%

Cardiovascular risk (diabetes mellitus, hypertension, hypercholesterolemia)	23.3%	61.8%	27.9%	10.8%	42.0%	56.2%

Body Mass Index < 25	65.1%	36.8%	35.6%	56.2%	36.1%	29.7%

25≤BMI < 30	27.6%	44.7%	45.0%	30.6%	40.3%	44.2%

BMI≥30	7.3%	18.5%	19.4%	13.2%	23.6%	26.2%

Smoker	38.4%	25.6%	7.9%	36.2%	32.0%	12.8%

Ex-smoker	15.7%	25.2%	23.5%	13.7%	22.9%	24.9%

Risk alcohol intake	6.1%	3.5%	1.5%	1.2%	2.0%	0.9%

We performed a numerical and graphic description of the prevalence of reported problems (moderate or extreme) in each dimension by comparing each age-sex group in Catalonia and the Canary Islands. Age was categorized in three groups: young adults (16-45 years), middle age (46-64 years) and seniors (65 and older). T-tests of proportions were made to compare prevalence of problems in both regions, for each age-sex category.

We then estimated a model to predict the EQ-5D-based HRQOL for each age-sex group in Catalonia and the Canary Islands, respectively. The model assumes that there is a latent health variable (y*) that is unobservable (the individual's real health) and depends on a combination of explanatory variables. Since the dependent variable is unobservable and we measure an ordinal proxy of it, an ordered probit is a suitable empirical model [20-22].

The dependent variable in our case is based on the subjective evaluation of the individual's general health with the EQ-5 D questionnaire. Our ordinal dependent variable (y) has four levels: the best possible state of health (y = 1) for those without any of the problems covered by the 5 dimensions of the EQ-5D; fair/good (y = 2) for those with a single moderate health problem in any of the 5 dimensions; fair/bad (y = 3) for those with two or three moderate problems; bad or very bad (y = 4) for those with more than three moderate problems or any extreme problem.

Our latent dependent variable model is

(1) $$y*=\beta^{'}X+\varepsilon$$

where y* is the unobservable continuous HRQOL, yet is identifiable, *X *is a vector of explanatory variables, *β *is a vector of coefficients of the factors that may affect health-related quality of life and *ϵ *is a random variable with a normal distribution.

The ordered probit model is based on the following expressions relating y* and y:

(2) $$\begin{array}{l} y=1↔y*\leq \mu_{1}\\ y=2↔\mu_{1}<y*\leq \mu_{2}\\ y=3↔\mu_{2}<y*\leq \mu_{3}\\ y=4↔\mu_{3}<y*\leq \mu_{4} \end{array}$$

where μ are unknown threshold parameters.

Explanatory variables are classified as follows: a) sociodemographic variables, b) health problems and use of healthcare services (diagnosed diseases, hospitalizations), and c) lifestyle variables. We also controlled for other potential confounders (unreported in the result tables), including occupation status and opinion of healthcare system. Our model can be expressed by the following equation:

$$y*=\beta_{0}+\beta_{1}X_{SD}+\beta_{2}X_{HEALTH}+\beta_{3}X_{LS}+\varepsilon$$

Our ordinal probit models were estimated by maximum likelihood. We performed post-estimation tests for significant differences between Catalonia and the Canary Islands in the impact of the diseases included on the HRQOL. We also tested whether educational level and risk factors influence health equally in both regions. The post-estimation tests were based on likelihood ratios between two competing models, a restricted one as null hypothesis (coefficients are equal) and an unrestricted one as alternative hypothesis. We fixed the significance level at 5%.

## Results

**Figure 1:**
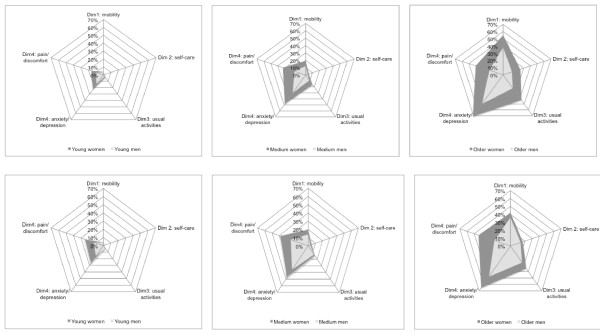
**Prevalence of health problems related to five dimensions (EQ-5D) in Catalonia and the Canary Islands by sex and age**.

Figure 1 shows the prevalence of problems in each dimension of the EQ-5 D, differentiating by gender, age group (16-44, 45-65 and 65 years or more) and the two regions.

There are substantial differences between gender, age groups and regions. The prevalence of problems in the five EQ-5 D dimensions increases with age and is generally higher for women than for men. Canarians have a higher prevalence of problems than Catalans, with the exception of mobility and depression problems for the group age 65 and above. The schedule dimension indicating the highest prevalence of problems in both regions is "anxiety/depression", ranging from 15% in the younger male Catalans (16% in the Canary Islands) to 71% in the older female Catalans (65% in the Canary Islands). The extent of discomfort and pain is noteworthy among Canary Island women: 25% of those young, 39% of the middle-aged and 44% of the elderly. T-tests of proportions show that the prevalence of problems in the four first dimensions is higher for old women in Catalonia than in Canary Islands (p < 0.001). On the contrary, prevalence of problems is higher in Canary Islands for middle-age men in dimensions 3, 4 and 5, and for all categories except old men for dimension 5 (p < 0.05)

A higher percentage of people declare themselves in good health in Catalonia than in the Canary Islands. The percentage of those who say their health is bad is similar for both regions. The degree of disparity of perceived health among inhabitants within each region is much greater. For instance, in Catalonia only 3.1% of young men declare to have bad health versus 40.1% of old women. Good health ranges from 18% (old women) to 78.4% (young men).

Tables 3, 4 and 5 present the results of the ordered probit models, expressed in marginal effects on the probability of belonging to the heath group. They also show whether there is a statistically significant difference from the reference category, in our study an individual (man or woman, Canarian or Catalan, as the case may be) who has completed elementary school, has a Body Mass Index of less than 25, and has no diagnosed chronic illness. We also controlled for the variables smoker, former smoker, and risk drinker, but they were not significant and the results are not included in the tables.

**Table 3:** Results of the ordered probit model for young adults (16-45)

CATALONIA 2006	Young women (N = 3696)				Young men (N = 3889)			
	Good health	Fair/good health	Fair/bad health	Bad health	Good health	Fair/good health	Fair/bad health	Bad health
No completed studies	0.0036	-0.0020	-0.0010	-0.0005	-0.0176	0.0119	0,0042	0,0015
Secondary school education	-0.0007	0.0004	0.0002	0.0001	0.0200	-0.0136	-0,0047	-0,0016
University education	**0.0487**	-0.0282	**-0.0139**	**-0.0066**	0.0158	-0.0109	-0,0037	-0,0012
Vascular problems	**-0.1053**	**0.0538**	**0.0328**	0.0187	-0.0371	0.0248	0,0091	0,0033
Osteomuscular problems	**-0.2202**	**0.1148**	**0.0673**	**0.0381**	**-0.2203**	**0.1362**	**0,0589**	**0,0252**
Respiratory problems	-0.0291	0.0161	0.0086	0.0044	-0.0262	0.0176	0,0063	0,0022
Digestive problems	**-0.1515**	**0.0739**	**0.0484**	**0.0293**	**-0.0876**	**0.0564**	**0,0225**	**0,0087**
Mental illnesses (depression/anxiety)	**-0.3040**	**0.1319**	**0.1007**	**0.0713**	**-0.3233**	**0.1710**	**0,0981**	**0,0542**
Cardiovascular risk	**-0.0526**	**0.0292**	**0.0155**	**0.0079**	**-0.0344**	**0.0232**	0,0083	0,0029
Overweight (25≤BMI < 30)	-0.0233	0.0130	0.0069	0.0034	0.0136	-0.0094	-0,0032	-0,0011
Obesity (BMI≥30)	0.0056	-0.0032	-0.0016	-0.0008	-0.0059	0.0040	0,0014	0,0005
								
Prob Y	72.12%	20.09%	5.80%	1.98%	82.13%	14.10%	3,01%	0,75%
	LR chi2(28) = 1120.13; Pseudo R2 = 0.1664				LR chi2(28) = 943.35; Pseudo R2 = 0.1736			
**CANARY ISLANDS 2004**	**Young women** **(N =1124)**				**Young men** **(N = 886)**			
	**Good** **health**	**Fair/good** **health**	**Fair/bad** **health**	**Bad** **health**	**Good** **health**	**Fair/good** **health**	**Fair/bad** **health**	**Bad** **health**
No completed studies	-0.0126	0.0048	0.0041	0.0038	0.0258	-0.0149	-0.0066	-0.0043
Secondary school education	0.0058	-0.0022	-0.0019	-0.0017	0.0115	-0.0066	-0.0029	-0.0020
University education	**0.0884**	**-0.0360**	**-0.0280**	**-0.0243**	0.0493	-0.0287	-0.0125	-0.0081
Vascular problems	0.0120	-0.0046	-0.0039	-0.0035	-0.0305	0.0169	0.0080	0.0056
Osteomuscular problems	**-0.1561**	**0.0510**	**0.0512**	**0.0539**	**-0.2159**	**0.1044**	**0.0604**	**0.0512**
Respiratory problems	-0.0220	0.0081	0.0071	0.0067	-0.0131	0.0073	0.0034	0.0023
Digestive problems	-0.0760	0.0264	0.0248	0.0247	-0.0403	0.0223	0.0106	0.0074
Mental illnesses (depression/anxiety)	**-0.3643**	**0.0728**	**0.1176**	**0.1739**	**-0.3657**	**0.1381**	**0.1070**	**0.1206**
Cardiovascular risk	0.0242	-0.0095	-0.0078	-0.0069	-0.0183	0.0103	0.0048	0.0033
Overweight (25≤BMI < 30)	-0.0244	0.0091	0.0079	0.0073	-0.0405	0.0228	0.0106	0.0072
Obesity (BMI≥30)	**-0.1357**	**0.0426**	**0.0447**	**0.0484**	**-0.1508**	**0.0763**	**0.0415**	**0.0330**
								
Prob Y	60.15%	24.36%	9.83%	5.67%	77.08%	16.17%	4.56%	2.19%
	LR chi2(22) = 260.10; Pseudo R2 =0.1072				LR chi2(23) = 145.51; Pseudo R2 = 0.1045			

In bold: significant at 5%

**Table 4:** Results of the ordered probit model for the middle-aged (46-64)

CATALONIA 2006	Mature women (N = 2166)				Mature men (N = 2155)			
	Good health	Fair/good health	Fair/bad health	Bad health	Good health	Fair/good health	Fair/bad health	Bad health
No completed studies	-0.0192	0.0012	0.0095	0.0085	-0.0050	0.0022	0.0019	0.0009
Secondary school education	**0.0578**	-0.0050	**-0.0284**	**-0.0244**	**0.0864**	**-0.0387**	**-0.0321**	**-0.0156**
University education	**0.0791**	-0.0106	**-0.0386**	**-0.0300**	0.0178	-0.0081	-0.0066	-0.0032
Vascular problems	**-0.0982**	-0.0013	**0.0483**	**0.0512**	0.0050	-0.0022	-0.0019	-0.0009
Osteomuscular problems	**-0.3364**	**0.0544**	**0.1577**	**0.1242**	**-0.3228**	**0.1300**	**0.1227**	**0.0701**
Respiratory problems	-0.0451	0.0018	0.0223	0.0211	-0.0170	0.0074	0.0064	0.0032
Digestive problems	**-0.1549**	-0.0073	**0.0754**	**0.0869**	**-0.0850**	**0.0346**	**0.0328**	**0.0176**
Mental illnesses (depression/anxiety)	**-0.3181**	-0.0148	**0.1484**	**0.1845**	**-0.3368**	**0.0936**	**0.1377**	**0.1056**
Cardiovascular risk	**-0.0810**	**0.0086**	**0.0397**	**0.0328**	**-0.0716**	**0.0321**	**0.0266**	**0.0129**
Overweight (25≤BMI < 30)	-0.0428	0.0028	0.0211	0.0189	0.0151	-0.0067	-0.0056	-0.0028
Obesity (BMI≥30)	**-0.0975**	0.0012	**0.0480**	**0.0483**	0.0084	-0.0037	-0.0031	-0.0015
								
Prob Y	37.92%	31.45%	21.56%	9.07%	63.49%	23.77%	9.72%	3.03%
	LR chi2(27) = 1303.60; Pseudo R2 = 0.2299				LR chi2(27) = 1038.06; Pseudo R2 = 0.2249			
**CANARY ISLANDS 2004**	**Mature women** **(N = 616)**				**Mature men** **(N = 445)**			
	**Good** **health**	**Fair/good** **health**	**Fair/bad** **health**	**Bad** **health**	**Good** **health**	**Fair/good** **health**	**Fair/bad** **health**	**Bad** **health**
No completed studies	-0.0467	-0.0005	0.0176	0.0297	0.0359	-0.0106	-0.0129	-0.0123
Secondary school education	**0.1399**	-0.0104	-0.0557	**-0.0738**	0.1303	-0.0453	-0.0459	-0.0391
University education	0.0796	-0.0029	-0.0311	-0.0457	0.0274	-0.0083	-0.0099	-0.0092
Vascular problems	-0.0609	-0.0016	0.0226	0.0399	-0.0540	0.0146	0.0196	0.0198
Osteomuscular problems	**-0.1985**	0.0002	**0.0742**	**0.1242**	**-0.2632**	**0.0596**	**0.0947**	**0.1088**
Respiratory problems	**-0.1313**	-0.0150	**0.0430**	0.1032	-0.1063	0.0236	0.0388	0.0439
Digestive problems	**-0.1117**	-0.0061	**0.0399**	**0.0779**	**-0.1445**	**0.0326**	**0.0527**	**0.0592**
Mental illnesses (depression/anxiety)	**-0.1896**	-0.0138	**0.0652**	**0.1383**	**-0.2901**	**0.0339**	**0.1029**	**0.1532**
Cardiovascular risk	0.0163	0.0001	-0.0062	-0.0103	**-0.1164**	**0.0316**	**0.0421**	**0.0427**
Overweight (25≤BMI < 30)	-0.0212	-0.0003	0.0080	0.0135	0.0223	-0.0065	-0.0080	-0.0077
Obesity (BMI≥30)	**-0.1125**	-0.0066	**0.0400**	**0.0792**	0.0531	-0.0166	-0.0190	-0.0175
								
Prob Y	37.27%	24.33%	22.81%	15.60%	52.64%	27.13%	12.97%	7.26%
	LR chi2(22) = 196.82; Pseudo R2 = 0.1199				LR chi2(23) = 196.71; Pseudo R2 = 0.1861			

In bold: significant at 5%

**Table 5:** Results of the ordered probit model for the elderly (65 and older)

CATALONIA 2006	Senior women (N = 1731)				Senior men (N = 1380)			
	Good health	Fair/good health	Fair/bad health	Bad health	Good health	Fair/good health	Fair/bad health	Bad health
No completed studies	**-0.0486**	**-0.0319**	0.0038	**0.0767**	-0.0259	0.0005	0.0147	0.0108
Secondary school education	0.0093	0.0060	-0.0009	-0.0144	**0.0868**	-0.0063	**-0.0488**	**-0.0317**
University education	0.0185	0.0114	-0.0023	-0.0276	**0.2004**	**-0.0342**	**-0.1086**	**-0.0576**
Vascular problems	**-0.0691**	**-0.0512**	-0.0024	**0.1227**	**-0.0587**	0.0001	**0.0332**	**0.0254**
Osteomuscular problems	**-0.2398**	**-0.0838**	**0.0784**	**0.2452**	**-0.3048**	**0.0240**	**0.1665**	**0.1143**
Respiratory problems	**-0.0454**	**-0.0341**	-0.0018	**0.0814**	**-0.0697**	-0.0012	**0.0395**	**0.0314**
Digestive problems	**-0.0514**	**-0.0371**	-0.0001	**0.0887**	**-0.0570**	-0.0003	**0.0323**	**0.0250**
Mental illnesses (depression/anxiety)	**-0.1345**	**-0.0985**	-0.0075	**0.2404**	**-0.2821**	**-0.0602**	**0.1420**	**0.2002**
Cardiovascular risk	**-0.0591**	**-0.0330**	0.0110	**0.0811**	-0.0349	0.0014	0.0197	0.0137
Overweight (25≤BMI < 30)	-0.0119	-0.0080	0.0008	0.0190	0.0165	-0.0004	-0.0093	-0.0067
Obesity (BMI≥30)	**-0.0566**	**-0.0427**	-0.0027	**0.1019**	-0.0593	-0.0009	0.0336	0.0266
								
Prob Y	13.96%	20.75%	34.42%	30.87%	36.51%	29.46%	25.67%	8.35%
	LR chi2(23) = 751.42; Pseudo R2 = 0.1609				LR chi2(23) = 626.26; Pseudo R2 = 0.1718			
**CANARY ISLANDS 2004**	**Senior women** **(N = 527)**				**Senior men** **(N = 325)**			
	**Good** **health**	**Fair/good** **health**	**Fair/bad** **health**	**Bad** **health**	**Good** **health**	**Fair/good** **health**	**Fair/bad** **health**	**Bad** **health**
No completed studies	**-0.2027**	**-0.0369**	**0.0496**	**0.1899**	**-0.2577**	0.0312	**0.1061**	**0.1204**
Secondary school education	-0.0792	-0.0347	-0.0018	0.1157	-0.0340	-0.0005	0.0134	0.0211
University education	0.0985	0.0223	-0.0213	-0.0995	-0.1520	-0.0186	0.0531	0.1174
Vascular problems	-0.0051	-0.0017	0.0006	0.0062	**-0.1062**	0.0001	**0.0421**	**0.0639**
Osteomuscular problems	**-0.1714**	**-0.0415**	**0.0332**	**0.1797**	**-0.2579**	0.0016	**0.1001**	**0.1561**
Respiratory problems	**-0.1008**	-0.0468	-0.0064	0.1541	-0.1242	-0.0102	0.0458	0.0887
Digestive problems	**-0.0741**	**-0.0270**	0.0054	**0.0957**	-0.0790	-0.0017	0.0309	0.0497
Mental illnesses (depression/anxiety)	**-0.1449**	**-0.0571**	0.0035	**0.1984**	-0.0888	-0.0044	0.0339	0.0593
Cardiovascular risk	**-0.0701**	**-0.0213**	0.0099	**0.0816**	-0.0562	0.0009	0.0226	0.0327
Overweight (25≤BMI < 30)	-0.0130	-0.0043	0.0014	0.0158	-0.0575	0.0007	0.0230	0.0337
Obesity (BMI≥30)	-0.0315	-0.0111	0.0029	0.0397	-0.0626	-0.0011	0.0246	0.0391
								
Prob Y	21.39%	19.11%	28.24%	31.26%	37.77%	25.95%	22.29%	13.99%
	LR chi2(17) = 133.68; Pseudo R2 = 0.0935				LR chi2(18) = 94.87; Pseudo R2 = 0.1098			

In bold: significant at 5%

Table 6 shows the results of the post-estimation tests for the hypothesis of equality of the coefficients of each variable or group of variables between the Catalan and Canary populations. Since we estimated models separately by age and sex, these tests are not affected by demographic differences between the two regional populations. Those tests show that the regional context is a significant factor, particularly for young and middle-aged women. The table shows the coefficients that are significantly different between Catalonia and Canary Islands at 5% of significance level. For men, the only disease that shows significant differences between the two regions in its effect on quality of life is mental illness among the elderly. Obesity is identified differently as a health problem only among young men from both regions.

**Table 6:** Contextual effects: LR Tests of equality between Catalonia and the Canary Islands.

		Young (16-44)		Middle-aged (45-64)		Old (65 and higher)	
		
		Women n = 4820	Men n = 4775	Women n = 2782	Men n = 2600	Women n = 2242	Men n = 1702
Education							**P = 0.025**

Diagnosed diseases	Vascular	**P = 0.028**				**P = 0.003**	
	
	Osteomuscular	**P = 0.008**		**P = 0.003**			
	
	Respiratory						
	
	Digestive						
	
	Mental			**P = 0.002**			**P = 0.002**
	
	All the above	**P = 0.002**		**P = 0.000**		**P = 0.001**	

Cardiovascular risk		**P = 0.05**		**P = 0.022**			

Hospitalization							

Obesity and overweight (BMI)			**P = 0.035**				

P-values of 5% significant differences

Blank = not significant (p value > 5%)

Level of education has a positive influence on health-related quality of life, and more so among elderly Canary Islands citizens of both sexes than among Catalonia citizens. Young women who have attended university are more likely to say they are healthy than those who have completed only primary school (8.9% more for Canarians, 4.9% more for Catalans). Among young men there is no significant difference by level of education. Among the middle-aged, those with secondary education reported better health than those with primary schooling only, with the exception of Canary men. Canary Island men and women who were elderly and had no schooling were at a considerable disadvantage compared to those who had completed at least primary school. In Catalonia the effect of education is much weaker. There are substantial structural and compositional differences between the two regions under consideration. 80% of the elderly in the Canary Islands did not complete their primary education compared to 46% of the elderly in Catalonia. The percentage of persons 65 and over who have attended university is similar (5.3% vs. 4.3%), but in primary and secondary schooling there are substantial differences in favor of Catalonia. But in addition to these compositional differences, the estimated influence of education on perceived health-related quality of life for elderly persons is significantly different in the Canary Islands than in Catalonia. There is a considerable number of elderly persons in the Canary Islands, as opposed to Catalonia, who are functionally illiterate, and this is an especial handicap in terms of health. Elderly Canarians of both sexes without studies are 20% to 25% less likely to be in good health than those who completed primary school. In Catalonia, in contrast, the effect is much smaller.

Having a diagnosed disease is clearly associated with a worse state of perceived health. The greatest correlation holds for mental illness, which is related to the anxiety/depression questions on the EQ-5 D, and for osteomuscular diseases that cause pain, as pain is one of the EQ-5 D questions. The association between diagnosed mental illness and poor self-reported health is generally greater in men than in women of the same age-group (with the exception of elderly Canarians), and overall less intense for old people, as if the older one gets, the more one learns to live with the illness. Hence among young males with diagnosed mental illness, the probability of reporting an optimal state of health is 37% lower for Canarians and 32% lower for Catalans than the reference category, which has no diagnosis of chronic illness. Among middle-aged persons with diagnosed mental illness, the probability of reporting an optimal state of health is 29% lower in Canary men (19% lower in Canary women) and 34% lower in Catalan men (32% lower in Catalan women). Elderly Catalan men diagnosed with mental illness are much less likely (28% less) to report an optimal state of health than elderly Canarian women (14%) and men (8%). Having a diagnosed osteomuscular disease has negative effects on self-rated health that are statistically significant with high coefficients in all population groups. The probability of reporting good health is reduced by a factor of 15% to 34%, depending on gender, age group and region. This negative effect is greater than that for mental illness for the elderly of both sexes, and for middle-aged women. Vascular diseases affect the self-reported health of Catalan women of all ages and that of elderly Catalan men, but have no significant effect on that of any population group in the Canary Islands.

As for cardiovascular risk, which includes high blood pressure, high cholesterol, and or diabetes, its effect on perceived health is low but statistically significant in young people in Catalonia, but not as strong as diagnosed mental illness or osteomuscular disease, and it has no effect on young Canarians. Overall the effect increases slightly in middle age and declines with old age. Obesity (Body Mass Index over 30) adversely affects the health-related quality of life of some population groups, but merely being overweight does not. Young Canarians of both sexes who report themselves as obese are less likely to say they are healthy. In Catalonia this effect is not significant for young people. In middle age obesity is significant only for women, and more so in the Canary Islands than in Catalonia. Among persons 65 or older the effect of obesity on perceived health is less important, and statistically significant only among Catalan women. The impact of respiratory diseases on quality of life is significant above all in old age, and more so among women, reaching the level of a 10% reduction in the probability of good health among Canary women over 65. Digestive problems have more impact on reported quality of life in middle age (and among young Catalans) than in old age.

## Discussion

The results demonstrate differences in perceived health between the two regions and between subgroups in each region. A higher percentage of people declare themselves in good health in Catalonia than in the Canary Islands (58% vs. 55%). The percentage of those who say their health is bad is similar for both regions (13%). But the degree of disparity of perceived health among inhabitants within each region is much greater. For instance, in Catalonia only 3.1% of young men declare to have bad health versus 40.1% of old women. Good health ranges from 18% (old women) to 78.4% (young men).

The explanatory variables of the ordinal probit model have different effects on health-related quality of life for the different age-groups and regions, but not all these differences are significant. Generally speaking, osteomuscular diseases and mental illness are the two kinds of health problems with the greatest impact on HRQOL, in part because the EQ-5 D questionnaire used has two dimensions (pain and anxiety/depression) closely associated with the effects of these diseases. Public health policies aimed at preventing these kinds of illnesses would have a disproportionate positive impact on quality of life.

The effect of education on health-related quality of life, especially among the elderly, is quite important. In addition to the well-known effects of education on health, on which there is an ample literature, it should be noted that since there is no data on individual or family income among the explanatory variables of our model, education may be reflecting in part the positive impact of income on perceived health. Old women in both regions report themselves unhealthier than men in the same age group, which may reflect the fact that chronic conditions are more prevalent among women, as a recent study has shown for Catalonia [23].

Although the surveys were carried out two years apart, we assume that the underlying processes that influence health and health parameters are comparable. Between 2004 and 2006 no in-deep health policies or interventions were made in Spain that could confound comparative results.

In regard to the comparison between Catalonia and Canary Islands, one important finding is that the effects of context are manifest above all in women; that is, that diagnosed chronic illness affects unequally the health-related quality of life of Catalan and Canary Island women. The explanation of that phenomenon is beyond the scope of this paper. For men, the only disease that shows significant differences between the two regions in its effect on quality of life is mental illness among the elderly, with much less impact on the Canary Islanders. It may be that among elderly Canary Islanders there is more tolerance for mental illness. Another surprising result is that obesity is considered quite problematic by young people in the Canary Islands, but not at all by those in Catalonia. One might speculate that this effect may be related to attractiveness in the critical period of courtship and the early years of marriage, but why this would be so in the Canary Islands but not in Catalonia may be due to regionally specific cultural differences, or changing international trends in the appreciation of body phenotypes that have reached one region sooner than another. Neither respiratory or digestive disease lead to regional differences in their effects on perceived health

All in all, the compositional/structural differences between Catalonia and the Canary Islands, as measured by comparison of the coefficients of each explanatory variable for different age-sex groups, are quite significant.

This study has its limitations. The most important is that we are using cross-sectional data. That is, we have a single observation for each person. Hence we cannot capture the effect of lifestyles on health, as in studies that use longitudinal data[24,25] because our cross-sectional data could reflect an inverse causality. An example of this inverse causality is that the health of smokers worsens gradually until there comes a time when they have to stop smoking. With cross-sectional data we may observe a positive relation between smoking and good self-assessed health because ill persons cannot smoke. It is not that smoking makes people healthy, but only that healthy people are able to go on smoking. For this reason, although we included in the regressions smoking and excessive consumption of alcohol as explanatory variables, these variables were not statistically significant and so are not shown in the tables. Obviously we cannot infer that these behaviors have no effects on people's health; but in order to examine their impact one would need longitudinal (panel) data.

There are additional relevant variables[26,27] that we have not been able to include in the analysis, whether because they are difficult to measure (the quality of medical care), or because they were not present in both databases (the Canary Islands survey does not provide information on personal and household income).

The variables that we had to work with had some drawbacks as well. Illnesses were self-reported, and while the subjects were asked about diseases that had been diagnosed or confirmed medically, their replies will have been affected by the availability or accessibility of medical services, which might well be quite different in the two regions. This factor could be especially relevant for diagnosed vascular diseases and cardiovascular risk factors.

The surveys we analyzed provide an overall state of the health of individuals that combines self-perceived health status (HRQOL) and certain common chronic conditions (self-reported, but based on known medical diagnosis). However, we do not know whether our subjects suffered from less prevalent diseases like HIV or Alzheimer's Disease. In this sense, the information provided by Health Surveys should be supplemented with *ad hoc *studies of diseases with important health and social impact but lower rates of incidence and prevalence. In addition, our study, like the great majority of studies on the general population, does not include persons who were institutionalized.

Our model finds that the impact of osteomuscular and mental diseases on health-related quality of life is clearly greater than those of the other diseases examined. Similar results were found in other works that used EQ-5D [15,16,28]. Yet cardiovascular diseases and tumors are the principal causes of death in Spain (and if one includes early deaths one would add death by external causes, such as accidents). The effectiveness of public health policies, like that of Quality-Adjusted Life Years (QALYs), must be judged not just by quality of life, but also by quantity ("to add life to years and years to life" as the WHO puts it). Diseases that cause early death have a special importance in public health policy and in the public mind, because of the number of years lost.

Despite these limitations, the measurement of the HRQOL of the population of a country or region and the study of its evolution can be a useful tool for decision-makers. Self-perceived health status can complement the information reported by indicators of life expectancy and the incidence and prevalence of morbidity. A complete description of the health status of population can assist an efficient allocation of health care and social resources in order to satisfy social needs. Furthermore, a synthetic indicator that combines quality of life and life expectancy can facilitate the comparison between costs and consequences of health policies, like those that prevent infant obesity, restrict tobacco and alcohol consumption, and coordinate prevention of ischemic heart diseases, tumors, mental illness, diabetes mellitus and stroke, to mention some of the most recent policies promoted by the Spain's health authorities. In this sense, the measurement of health population using multidimensional concepts would lead to a better understanding of health care effectiveness and a better evaluation of health care returns.

## Conclusions

This study shows clear differences in the impact of a variety of health problems on the health related quality of life between men and women as between persons of different ages, regions and educational backgrounds. The findings show the importance of disease prevention and early detection of chronic conditions in order to enhance health-related quality of life, point to the need for broad policies to improve the educational level of the population in order to reduce health inequalities, and indicate that further research would improve our knowledge about explanatory variables that affect the quality of life of individuals though the life cycle.

## Abbreviations

SHR: Self Rated Health; HRQOL: Health-Related Quality Of Life; CHS: Health Survey of Catalonia; CIHS: Canary Islands Health Survey.

## Competing interests

The authors declare that they have no competing interests.

## Authors' contributions

JOM conceived of the study and participated in its design, the data acquisition, the data analysis, and the draft of the manuscript. NB and BGLV participated in the data analysis, and to the manuscript revision. All authors have read and approved the final manuscript.

## Pre-publication history

The pre-publication history for this paper can be accessed here:

http://www.biomedcentral.com/1471-2458/10/576/prepub
